# Combined T2 and diffusion-weighted MR imaging with template prostate biopsies in men suspected with prostate cancer but negative transrectal ultrasound-guided biopsies

**DOI:** 10.1007/s00345-016-1855-x

**Published:** 2016-05-28

**Authors:** Nissar Sheikh, Cheng Wei, Magdalena Szewczyk-Bieda, Annie Campbell, Shaukat Memon, Stephen Lang, Ghulam Nabi

**Affiliations:** 1 Division of Cancer Research, School of Medicine, University of Dundee, Ninewells Hospital, Dundee, DD1 9SY UK; 2 Department of Urology, Pilgrim Hospital, United Lincolnshire NHS Trust, England, UK; 3 Department of Clinical Radiology, Ninewells Hospital, Dundee, DD1 9SY UK; 4 Department of Pathology, Ninewells Hospital, Dundee, DD1 9SY UK; 5 Technology & Innovation in Learning Team (TILT), School of Medicine, University of Dundee, Dundee, DD1 9SY UK

**Keywords:** Prostate cancer (PCa), Transperineal template biopsy (TPB), Multiparametric MRI (mpMRI), Transrectal ultrasound (TRUS), Diffusion-weighted imaging (DWI), T2-weighted image (T2WI)

## Abstract

**Purpose:**

Transperineal template prostate (TPB) biopsy has been shown to improve prostate cancer detection in men with rising PSA and previous negative TRUS biopsies. Diagnostic performance of this approach especially MR imaging and using reliable reference standard remains scantly reported.

**Materials and methods:**

A total of 200 patients, who were previously TRUS biopsy negative, were recruited in this study. All the participants had at least 28-core TPB under general anesthetic within 8 weeks of previous negative TRUS biopsies. In 15 men undergoing laparoscopic radical prostatectomy, prostate specimens were sectioned using custom-made molds and analyzed by experienced pathologist as a feasibility study.

**Results:**

In total, 120 of 200 patients (60 %) had positive TPB biopsy results. All of these men had at least one negative biopsy from transrectal route. T2 diffusion-weighted MR imaging showed no lesion in almost one-third of these men (61/200; 30.5 %). Out of these, 33 (33/61; 54 %) showed malignancy on TPB including high-grade tumors (>Gleason 7). Out of 15 patients underwent surgery with a total of 52 lesions (mean 3.5) on radical prostatectomy histology analyses, TPB detected 36 (70 %) lesions only. Some of these lesions were Gleason 7 and more mostly located in the posterior basal area of prostate.

**Conclusions:**

Transperineal template biopsy technique is associated with significantly high prostate cancer detection rate in men with previous negative TRUS biopsies, however compared to radical prostatectomy histology map, a significant number of lesions can still be missed in the posterior and basal area of prostate.

## Background

Transrectal ultrasound (TRUS)-guided biopsy in men suspected of having prostate cancer has been an established clinical practice [1, 2]. Transperineal route of needle biopsies under guidance of a TRUS probe is emerging as a second line investigation in the diagnostic care pathway, particularly in men with previous negative biopsies and rising PSA [3]. The burgeoning interest in this approach is due to improved sampling of anterolateral part of the gland, better information on grade and volume of cancer and perhaps a reduced risk of infection compared to transrectal route [4, 5]. However, this approach is still offered in only a small percentage of patients, in particular in conjunction with pre-biopsy MRI, as shown by an audit carried out on behalf of Urological Society of Australia and New Zealand [6, 7]. Despite this, several previous studies have confirmed its usefulness in men with previous negative biopsies [4].

T2-weighted MR images provide useful information about prostate anatomy and help in visualization of cancer lesions in prostate [8]. MR imaging has been increasingly adopted in men with previous negative TRUS biopsies and rising PSA. Diagnostic accuracy of T2-weighted imaging (WI) varies between 50 and 90 % [9]. Also, data on methods of imaging and pathology co-orientation, in order to compare diagnostic accuracy of MR imaging with radical prostatectomy specimen, are lacking.

The recent technological development of rapid prototyping, a technique by which 2D images are processed into 3D computerized physical models, has provided clinicians and bioengineers with a new way to understand complex spatial relationships of prostate and potential biopsy-related procedural complexities more clearly [10–12]. This allows operators physical visualization and device–anatomy orientation in 3D space. Rapid prototyping represents a new technology, which is likely to play a significant role in the patient-specific planning of prostate and other oncological interventions.

There are several studies reporting advantages of transperineal template biopsies (TPB) [13–16]; however, the literature comparing its diagnostic accuracy in comparison with whole mount prostate specimen is scanty and mostly retrospective in nature [14]. There are no studies reporting on comparison of multiparametric MRI (mpMRI), template biopsies and detailed histology of prostatectomy specimen, processed in orientation with the MRI images using 3D printing and molds for patient-specific prostate.

In the present study, we investigated diagnostic accuracy of TPB in men with previous negative TRUS biopsies in particular men with negative MRI and positive TPB biopsies. A proof of concept study was also carried out, using patient-specific custom-built prostate molds and radical prostatectomy histology as a reference standard.

## Patients and methods

### Study population and patient recruitment

**Fig. 1:**
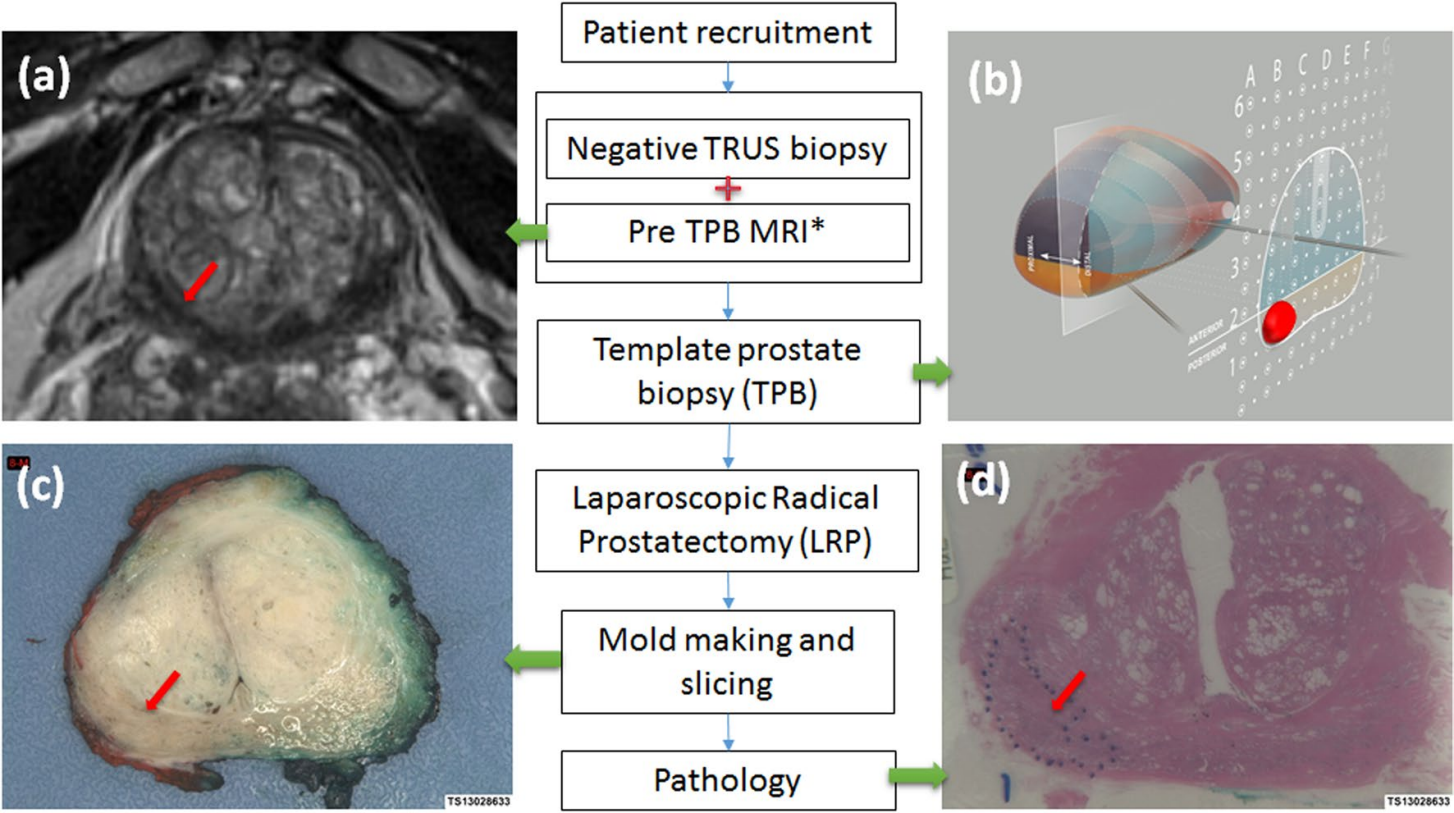
Flow chart of study. One of the prostate lesions shows in T2-weighted MR images (**a**, *red arrow*); template prostate biopsy (**b**, *red* *mass* *area*); prostatectomy specimen slice (**c**, *red arrow*); and histology photo (**d**, *red arrow*). *Asterisk:* Pre-TPB is within 8 weeks of previous negative TRUS biopsies

Between January 2013 and June 2015, 200 men with at least one previous negative TRUS biopsies and rising PSA were offered TPB in a multi-institutional setting. The study was registered as an audit project at both participating centers, and institutional approvals were obtained (Pilgrim Hospital audit number P2251/2015 and Ninewells Hospital, Caldicott, approval number CSAppGN021211). All men had MRI imaging of prostate prior to TPB. As a proof of concept, we selected fifteen men subsequently opting for radical prostatectomy (RP) for a detailed analysis of the prostate specimen. MRI images were used for 3D printing and custom-created patient-specific molds to enable histopathological processing of prostates after RP. Baseline characteristics of patients are shown in Table 1. Flow chart of the study is shown in Fig. 1.

**Table 1:** Characteristics of the study cohort

Number of patients (%)	200 (100 %)
Age, year, mean (range)	66 (48–78)
PSA level, ng/ml, median (range)	12.5 (8–36)
Prostate volume, cc, median (range)	45 (23–200)
Prostate size	
Left–right, mm, median (IQR)	55 (52–60)
Apex–base, mm, median (IQR)	42 (38–47)
Anterior–posterior, mm, median (IQR)	42 (38–47)
Number of previous negative TRUS biopsies (%)	
1	160 (80 %)
2	26 (13 %)
3	14 (7 %)

### MR imaging protocol

Most of the MR imaging in the participating centers was carried out on 1.5 T scanners (Siemens Medical Solutions), equipped with surface phased pelvic array (Body Matrix, Siemens Medical Solutions).

Anatomical imaging of the prostatic gland was obtained by acquiring turbo spin-echo (TSE) T2-weighted sequences in the axial, sagittal and coronal planes, with the use of optimized parameters for a better spatial resolution.

All patients underwent diffusion-weighted imaging (DWI), (2D echo-planar imaging spin-echo, with at least three b-values and calculated ADC map) in addition to anatomical T2WI.

The MR imaging was performed for staging purposes 6–7 weeks post-TRUS biopsy to minimize artifact from post-biopsy hemorrhage.

In a small subset of cohort meant for custom-created molds, MR imaging was carried out using 3T scanner (TIM Trio, Siemens Medical Solutions) using multiparametric (mp) approach, with standard protocol in accordance with European Society of Uro-radiology Guidelines 2012 [17]. The protocol combines anatomical sequences (TSE T2 and T1WI) with functional imaging, including DWI sequences with three b-values (0,400 and 1000 s/mm^2^) and a separate high b-value (2000 s/mm^2^) acquisition and dynamic contrast-enhanced (DCE) sequences (3D fast gradient-echo sequences with temporal resolution of 4 s, using 2 ml/kg of Dotarem, gadolinium-based contrast agent [Guerbet, France]).

The mpMR images were assessed and scored by experienced uro-radiologist without knowledge of histopathology.

In accordance with the European consensus meeting statement on mpMRI to detect, localize and characterize prostate cancer, the prostate images were divided into a 27 regions of interest (apical, mid and base quadrants) [18]. Regions, which contained suspected lesions, were marked as affected area for analyses and comparison with histopathology.

T2 and DWI parameters were assessed and lesions labeled as suspicious of prostate cancer prior to template biopsies. In 40 men, images were acquired using 3T MR machine and this includes those where radical prostatectomy specimen were oriented to imaging through mold fabrication process described below.

### Customized 3D printed molds of prostate specimen for histology

**Fig. 2:**
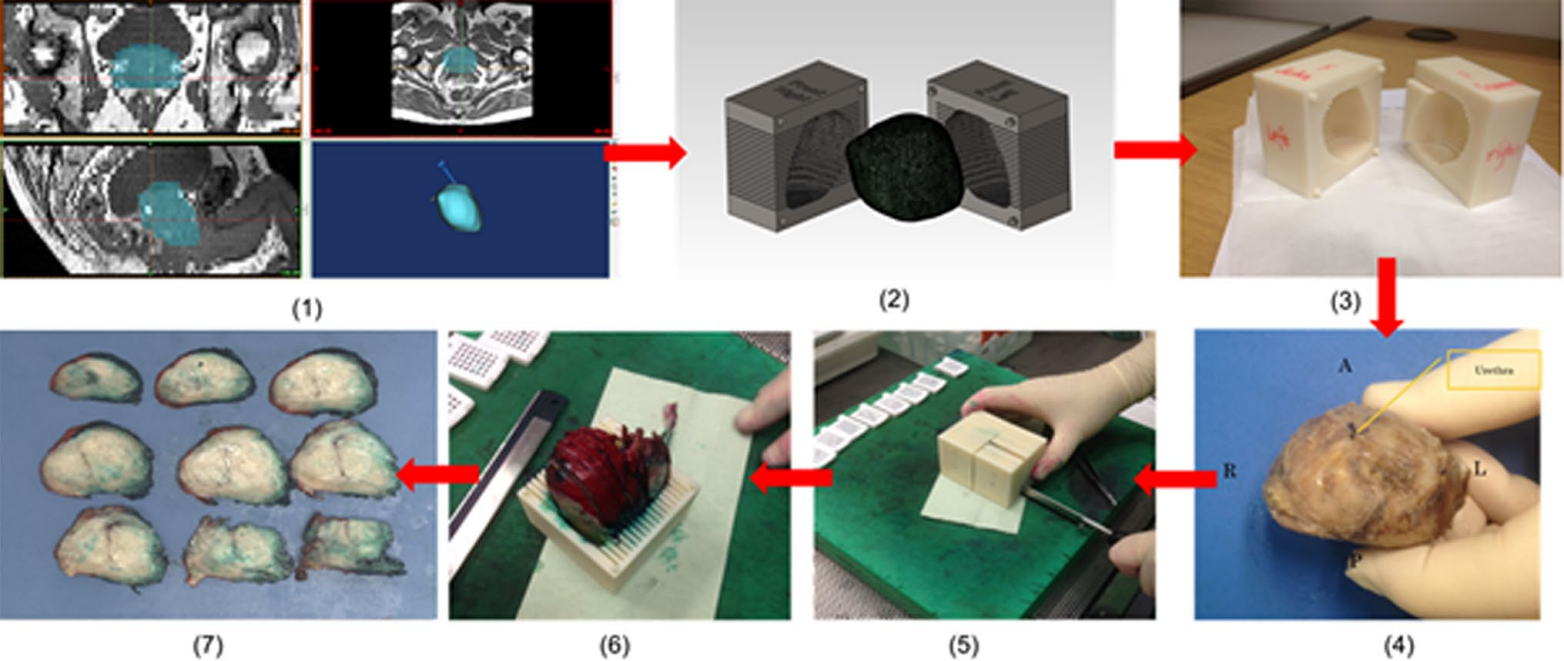
Steps of patient-specific molds fabrication and histopathological sectioning—**1** segmentation of MRI data in biomedical software MIMICS, **2** mold fabrication in CAD software SolidWorks, **3** 3D printout from rapid prototyping machine MakerBot, **4** post-radical prostatectomy specimen before dyeing and mold placement, **5** slicing of prostate specimen with a single blade, **6** sliced sections shown in the mold and **7** specimen slices arranged from apex to base

The process for patient-specific molds, based on anatomical MRI (T2WI) and prostate sectioning during histopathology processing, is illustrated in Fig. 2 followed by detailed description.

#### 3D segmentation of prostate contour

**Fig. 3:**
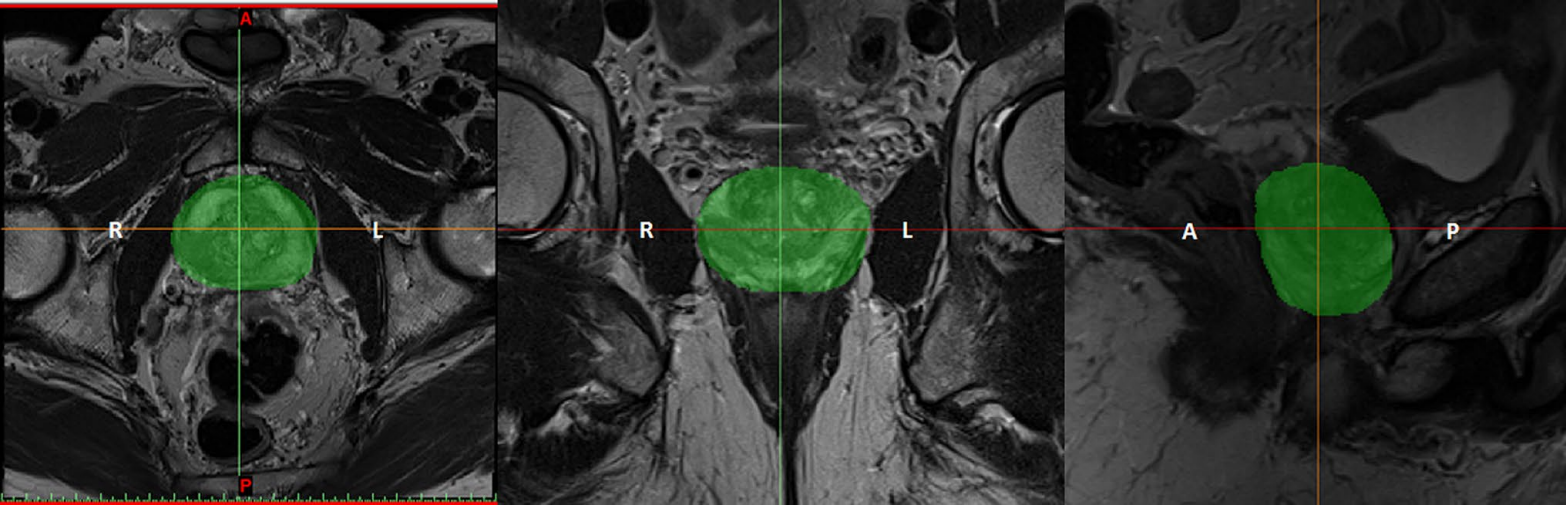
Segmentation of the prostate (*green* area) in three different views of MR imaging, from *left* to *right* axial, coronal and sagittal

Participants had 3T mpMRI scans before their preferred surgical treatment option (laparoscopic radical prostatectomy). Three planar views (axial, coronal and sagittal) of the prostate were obtained by T2-weighted images (T2WI); the thickness of each slice was 3 mm with 0.6 mm gap and the scan resolution for axial view of 0.63 × 0.63 mm^2^. After a detailed analysis of 2D pelvic images, the boundary of the prostate capsule was traced with the help of an experienced uro-radiologist and MIMICS software (Medical Image Segmentation for Engineering on Anatomy), and the segmentation of the prostate was done in one direction (mostly axial) and modified in the other two directions (as shown in Fig. 3). The model was saved as a stereolithography (STL) file.

#### 3D prostate model generation and modification

With the 9–12, T2WI in axial direction of segmentation, one 3D model was fused and converted into an object simulated in software MIMICS representing patient’s prostate topography. Before any modification, the 3D prostate model was verified and compared with the previous T2WI to avoid any possible misstatement, which made sure mold’s outline was matched with MR images. The capsule of the fused 3D model was fairly rough when verification was done so smoothing was applied on the surface of capsule. The coarse model was imported to CAD software Meshmixer (2011 Autodesk, Inc.) for the purpose of smoothing and reducing the difficulty of mold fabrication. The smoothed model was also saved in STL file as a mesh model and transferred into MIMICS again for triangle reduction to reduce the file size.

#### Mold design

The triangle-reduced mesh model was imported into SolidWorks (3D CAD design software, analysis software, Dassault Systèmes SolidWorks Corp. USA) as a SolidWorks Part (SLDPRT) model. For a successful conversion, the triangle or mesh surfaces of a model had to be controlled within a certain limit, and otherwise the importing process would have been influenced with a risk of collapse. The solid simulated prostate model was then wrapped up by a cubic or rectangular model block, and “subtraction” (combined in feature) was applied to the two models. As a result, a new model, with an internal cavity for precise holding of patient’s prostate, was created, according to individual patient’s MRI scans. The outside dimensions of the molds were varied because patients had different shapes and sizes of prostates. Once the position of prostate in the mold was determined, slots for sectioning the specimens were placed into the mold with a reasonable location of axial direction according to 2D images in axial plane MRI. Each slot was 1.2 mm thick for an easy cutoff with a single trimming blade with thickness of 0.245 mm (Feather Safety Razor Co., LTD. Medical Division) and with a 3-mm interval, which represented the thickness of each MRI axial slice. The orientation of the slots could also be sagittal and coronal, depending on the fusing direction of patients’ prostate model. The mold was then divided into 2 halves (left and right) using feature “split” for embedding of prostate specimen. The two separated parts, which were edited in SLDPRT files, were saved as STL files.

#### Mold prototyping with fused filament fabrication technology

3D fabrication of each mold was done in a 3D printer (MakerBot Replicator 2X). The 3D printer has dual print heads, which could create two different parts with fused deposition of materials at the same time. Acrylonitrile–butadiene–styrene (ABS) in 1.75 mm (filament diameter), which is made of a combination of those three plastics, is the type of printing material for Replicator 2X. The patients’ mold parts were printed layer by layer, and the layer height of extruded filament could be as small as 100 microns. All solid portions of the model parts were automatically printed in the lattice of honeycomb for reducing materials usage and print time. Printing time ranging from 4 to 7 h was dependant on the size of each mold and the applied layer height (from 100 to 300 microns). Before the final printing, the STL files of two mold halves were imported to software MakerWare to orient the printing position, calculate the approximate printing time and amount of ABS plastic needed. Rafts were also added on the bottom of the two mold halves to provide a better build surface and avoid any warping when layers closer to the extruder were cooling and shrinking

#### Specimen dyeing and sectioning

Once the prostate specimen was acquired from a patient after surgical removal, it was transferred to pathology laboratory and placed in formalin for 48–72 h at room temperature. Before being sliced in the mold, the specimen was painted so that the left part was green and right part was red, and then the seminal vesicles were excised as per mold from apex to base of prostate. When placing the prostate specimen into the mold, the right orientation needed to be checked so that the sliced histopathology specimens would represent their respective MRI scanning slices. During the sectioning, a single blade was used to cut the prostate and it had to be applied carefully and patiently to avoid specimen friction and shifting. After slicing, all histopathology sections were photographed and stored in separated tissue blocks for further analysis.

### Data analysis

Diagnostic accuracy for TPB was assessed for different regions and zones of the prostate as shown in Fig. 1. The histopathological sections of whole mount specimen were used as reference standard in a subset of cohort, as described in methods. Further analysis included diagnostic accuracy of TPB with regards to grade and size of cancer foci in relation to prostate specimen histology.

## Results

Mean age of all patients in a cohort was 66 years (range 48–78), mean prostate volume was 45 cc (range 23–200) and mean PSA was 12.5 ng/ml (range 8–36). All men had at least one negative TRUS biopsy.

120 (120/200; 60 %) of men were found to have prostate cancer confirmed by TPB.

In 139 (139/200; 69.5 %) men, MRI scan detected at least one suspicious lesion, detecting on average 1–3 lesions within the gland. Histopathology of TPB showed cancer in 84 patients (84/139; 60.4 %).

**Fig. 4:**
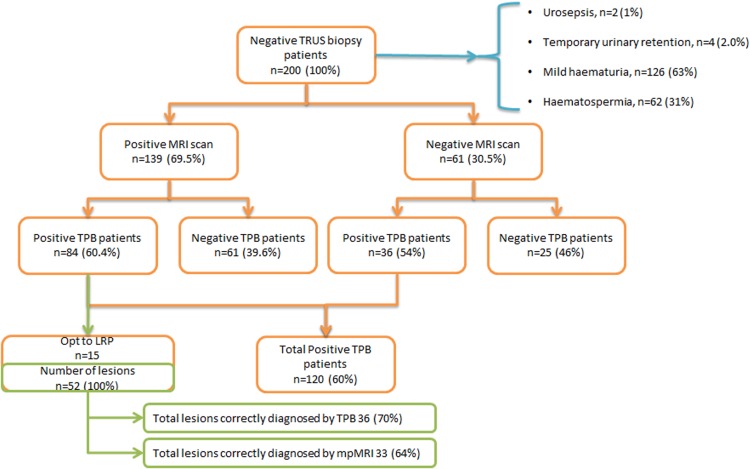
Patient outcome and lesion detection in different methods

**Fig. 5:**
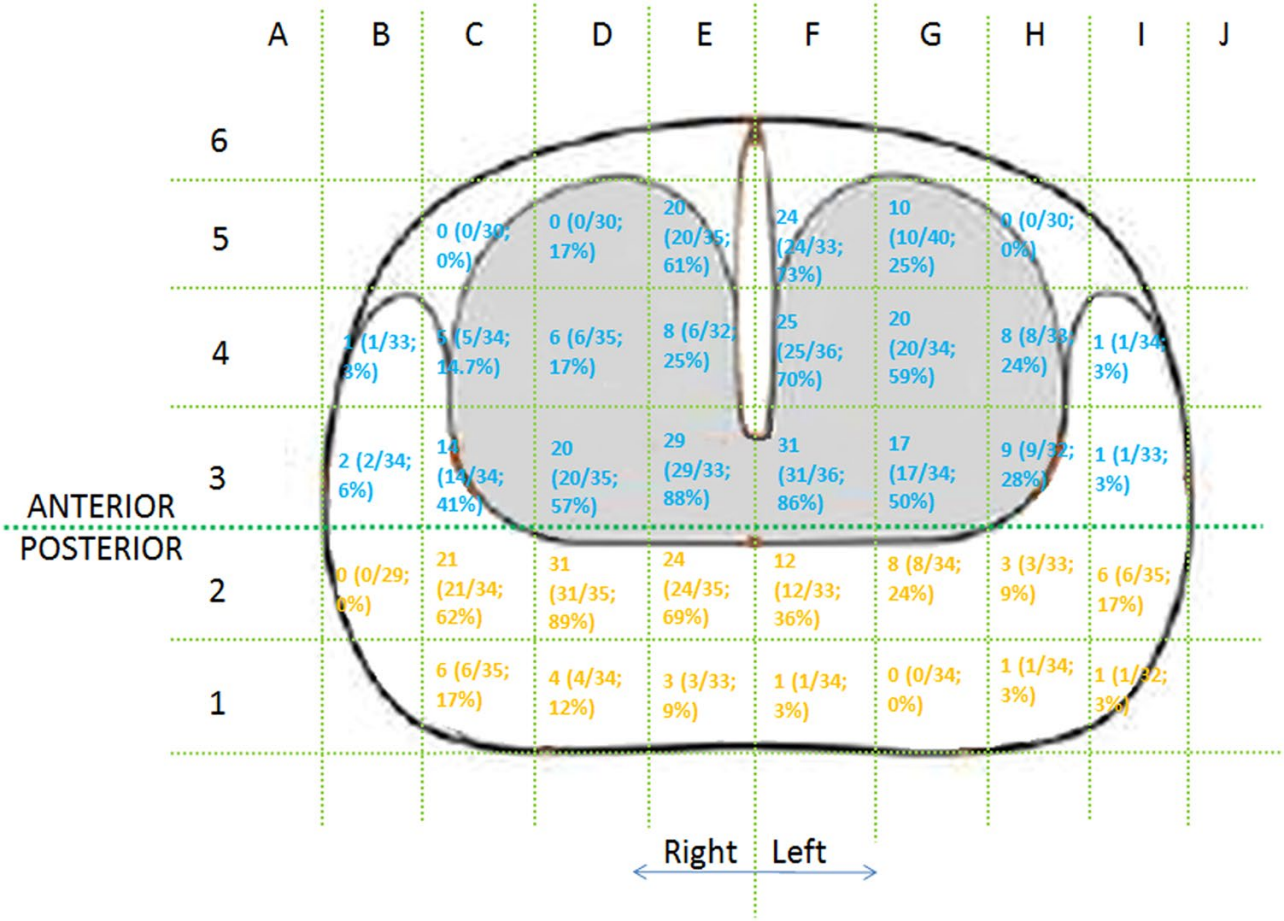
Distribution of cancer foci in 36 patients with negative MRI and positive TPB

In 61 (61/200; 30.5 %) patients, no definable lesions were seen in prostate on imaging (50 on 1.5 T and 11 on 3T machine). 36 (36/61; 59 %) of these men were shown to have prostate cancer on template biopsies(Fig. 4). Distribution of cancer foci is shown in Fig. 5. Some of these lesions were significant based on previously published criteria [19] [20] and patients required radical treatment .

In proof of concept study with orientation of imaging with histopathology, there were 52 lesions seen in 15 prostate specimens, ranging from size of 2 mm to 3 cm. 36 of these were correctly identified by TPB (70 %) (Fig. 4). Region-wise detection rate for TPB and location of suspected lesions on MRI are shown in Table 2. This demonstrates that most of the lesions missed by TPB are smaller and in the basal posterior region of prostate. Table 2 also shows correlation between size of lesions in different regions and diagnostic accuracy of TPB approach.

**Table 2:** Number of foci of prostate cancer (PIRAD score 3 or more) correctly detected by template biopsies (TPB) and multiparametric MRI (mpMRI) versus histopathology of laparoscopic radical prostatectomy (LRP)

Gleason grade	Size of lesions	mpMRI		TPB		LRP (reference)	
		Anterior	Posterior	Anterior	Posterior	Anterior	Posterior
Gleason 6	<5 mm	0 (0 %)	3 (50 %)	2 (67 %)	3 (50 %)	3	6
	≥5 mm	4 (80 %)	4 (100 %)	5 (100 %)	3 (75 %)	5	4
Gleason 7	<5 mm	2 (50 %)	3 (38 %)	3 (75 %)	5 (63 %)	4	8
	≥5 mm	6 (100 %)	7 (100 %)	6 (100 %)	5 (71 %)	6	7
Gleason 8 or more	<5 mm	0 (0 %)	0 (0 %)	1 (100 %)	1 (33 %)	1	3
	≥5 mm	1 (100 %)	3 (75 %)	0 (0 %)	2 (50 %)	1	4
Sub-total		13 (65 %)	20 (63 %)	17 (85 %)	19 (59 %)	20	32
Total		33 (64 %)		36 (70 %)		52	

## Discussion

The study showed that template biopsies of prostate detected cancer in 60 % of men with previous negative TRUS biopsies. The detection rate was higher if lesions were seen on MR imaging (60.4 vs. 54 %) compared to no definable abnormality on MR imaging. Interestingly, in more than half men with no definable lesions on 1.5 T MR imaging, cancers of various grades were detected on TPB. This highlights the fact that post-biopsy MRI cannot replace histopathology from biopsies as a diagnostic modality and area needs further research, using pre-biopsy mpMRI protocol, which shows promising detection rate for clinically significant cancers [21, 22]. It is quite possible that advanced multiparametric imaging using 3T machines in all the patients may have picked some of these foci, however not all centers, especially small district general and community hospitals have access to this technology.

Our findings contribute to filling the gap in knowledge as highlighted by National Institute of Health and Excellence (NICE) guidelines panel [23].

In the detailed, analysis using patient-specific molds, lesions in posterior location near the bases and those smaller than 5 mm are missed in almost one in five patients. Multiparametric MRI was used to locate abnormal foci in all these participants prior to TPB and interestingly performs slightly less than transperineal biopsies approach especially in anterior prostate region. In smaller lesions less than 5 mm, mpMRI did not perform well even for high-grade disease (31.3 % (5/16) in Gleason 7, 8 and more); however, it was much better in lesions more than 5 mm. The findings of the study confirm complementary role of advanced MR Imaging and improved sampling using TPB in better detection and localization of prostate cancer foci.

The study uses 3D printing, also known as rapid prototyping to provide an improved orientation between imaging and histopathology data of radical prostatectomy specimen which then facilitates assessing diagnostic accuracy of template biopsies. This requires a multi-disciplinary approach and combined effort of surgeon, bioengineer and radiologist. The real use of this technique in the present study was to support pathologist in correctly aligning histopathological slices of prostate with the MR imaging data. This approach improved our understanding of cancer foci distribution within the prostate gland and reliable comparison of what is seen in MRI and pathological sectioning. Patient-specific molds were created and made available to histopathologist prior to sectioning of prostate. The histopathologist was not aware of MRI findings while reporting Gleason score of number of cancer foci in the prostate specimen. In the future and more data available, 3D print may exist in pathology in routine clinical practices, especially when this involves a low-cost desktop 3D printer (Makerbot or Ultimaker) and manufactured molds using £0.6 worth of material over the course of 4–7 h, with minimal technical manpower input. Our study may facilitate this and future research in this area.

There is ongoing research and innovations to improve MR–histology correlation mainly driven by desire to translate predictive value of MRI into clinical practice. Although there are a number of factors responsible for variations in the reports of sensitivity and specificity of MR imaging in visualization and interpretation of tumors on MRI, correlation between MR–histology remains a major one [24, 25]. Spatial correlation using methodology of the present and previously described studies [11, 12] is a step toward addressing this issue.

The reported studies have suggested that the ex vivo shape of the prostate is considerably different from the in vivo contour [26, 27]. In the present study, surface pelvic coil was used compared to endorectal coil reported previously which compresses the prostate [28]. We have studied this issue (unpublished results), and there were no significant differences (8 % increase in volume of ex vivo prostate specimen) between in vivo prostate imaging and ex vivo prostate geometry; however, this remains a theoretical risk.

The present study also highlights poor performance of TRUS-guided biopsies in sampling disease even in peripheral zone close to rectal approach. Some of the previous studies suggest saturation biopsies as a second best step; however, such an approach is still not ideal for anteriorly located tumors. The emerging consensus, although with no strong evidence available yet, is that men with negative TRUS biopsies and rising PSA should be offered MR imaging for the detection of anteriorly placed prostate cancer should be kept in mind. The present study and our clinical practice reflect this. As pointed out above, complementary performance of both MR imaging and TPB should help in improving sampling and detection of prostate cancers both in anterior and posterior base regions. Although posterior area was much smaller than anterior portion in the transperineal biopsy template, there were 12 more lesions detected in posterior area. Both MRI and TPB had similar detection rate in posterior portion (63 vs. 59 %), which means more samples are needed in this part. Small lesions (<5 mm) were still hard for TPB to recognize but was far better than MRI. MRI had a higher accuracy in pointing out ≥5-mm lesions (93 vs. 78 %). Half of lesions were Gleason Grade 7; TPB detected 9 out of 10 lesions in anterior but only 10 out of 15 lesions in posterior.

There was more than one focus of cancers with histological heterogeneity in all the patients. Which cancer foci are significant and may ultimately be responsible for significant outcomes such as metastases and death? To answer this question, Heffner et al. [29] used whole-genome sequencing and molecular pathological analyses to characterize the lethal cell clone in a patient who died of prostate cancer. They tracked the evolution of the lethal cell clone from the primary cancer to metastases through samples collected during disease progression and at the time of death. Surprisingly, these analyses revealed that the lethal clone arose from a small, relatively low-grade cancer focus in the primary tumor, and not from the bulk, higher-grade primary cancer or from a lymph node metastasis resected at prostatectomy. This interesting but crucial question needs further research.

There was no correlation between the size of prostate gland and detection of prostate cancer, although admittedly the study was not designed to answer this question. Mean size of prostate in this series was 45 cc (range 23–200).

## Conclusions

Transperineal template biopsy technique is associated with significantly higher prostate cancer detection rate in men than previous negative TRUS biopsies, however compared to radical prostatectomy histology map, a significant number of lesions can still be missed in the posterior and basal area of prostate. Small but high Gleason Grade cancers are still a challenge for TPB although it has a higher performance than mpMRI, based on a small patient sample analyzed.
